# Structural and optical properties of ZnO nanorods by electrochemical growth using multi-walled carbon nanotube-composed seed layers

**DOI:** 10.1186/1556-276X-7-13

**Published:** 2012-01-05

**Authors:** Yeong Hwan Ko, Myung Sub Kim, Jae Su Yu

**Affiliations:** 1 Department of Electronics and Radio Engineering, Kyung Hee University, 1 Seocheon-dong, Giheung-gu, Yongin-si, Gyeonggi-do, 446-701, South Korea

**Keywords:** ZnO nanorod arrays, multi-walled carbon nanotubes, electrochemical growth, crystallinity, photoluminescence

## Abstract

We reported the enhancement of the structural and optical properties of electrochemically synthesized zinc oxide [ZnO] nanorod arrays [NRAs] using the multi-walled carbon nanotube [MWCNT]-composed seed layers, which were formed by spin-coating the aqueous seed solution containing MWCNTs on the indium tin oxide-coated glass substrate. The MWCNT-composed seed layer served as the efficient nucleation surface as well as the film with better electrical conductivity, thus leading to a more uniform high-density ZnO NRAs with an improved crystal quality during the electrochemical deposition process. For ZnO NRAs grown on the seed layer containing MWCNTs (2 wt.%), the photoluminescence peak intensity of the near-band-edge emission at a wavelength of approximately 375 nm was enhanced by 2.8 times compared with that of the ZnO nanorods grown without the seed layer due to the high crystallinity of ZnO NRAs and the surface plasmon-meditated emission enhancement by MWCNTs. The effect of the MWCNT-composed seed layer on the surface wettability was also investigated.

**PACS: **81.07.-b; 81.16.-c; 81.07.Pr; 61.48.De.

## Introduction

In the past decade, various ZnO nanostructures including nanowires, nanorods, nanosheets, nanoflowers, and nanotubes have received intensive attention because of their excellent physical properties for a wide range of practical device applications such as ultraviolet photodetectors, field-effect transistors, light-emitting diodes, and biological and chemical sensors [1-3]. Among many fabrication approaches, a hydrothermal or electrochemical deposition method has been considered as an efficient way to grow ZnO nanostructures since it is a simple, low-temperature, large-scale, and cost-effective process [4,5]. In order to grow high-quality ZnO nanostructures in such chemical synthesis methods, the seed layer is very important because the orientation and crystallinity of ZnO nanostructures depend on the conditions of the underlying seed layer [6]. For this reason, the radio frequency [rf] sputtering or atomic layer deposition and subsequent thermal annealing treatment have been employed to form a good seed layer with excellent step coverage, thickness controllability, and reproducibility. However, it requires a somewhat complicated procedure and high-vacuum environment.

Meanwhile, carbon nanotubes [CNTs] have been one of the most advanced functional materials because of their superior electronic property, good thermal/chemical stability, high mechanical strength, and large surface area [7-9]. Recently, the ZnO/CNT composites and hybrid nanostructures have been considered as a promising candidate for improving the device efficiency in the electronic and optoelectronic devices because these structures can provide the enhanced electrical and optical properties by the cooperative physical interaction between ZnO nanostructures and CNTs [10]. However, achieving good control over the size and morphology of the ZnO/CNT hybrid structures is still difficult. Thus, the investigation of the seed layer containing CNTs for growing the ZnO nanostructures is very interesting. In this work, the electrochemically synthesized ZnO nanorod arrays [NRAs] after spin-coating an aqueous seed solution containing MWCNTs, which can be expected to be a facile and efficient process for the fabrication of high-quality ZnO NRAs, were studied. Their structural and optical properties were also evaluated.

## Experimental details

All chemicals were purchased from Sigma-Aldrich Corporation (St. Louis, MO, USA) and Kojundo Chemical Laboratory Co., Ltd. (Saitama, Japan), which were of analytical grade and used without further purification. The MWCNTs and indium tin oxide [ITO]-coated soda-lime glasses were also purchased from Hanwha-Nanotech (Incheon, South Korea) and Samsung Corning (Seoul, South Korea), respectively. The ITO coated on the soda-lime glass (i.e., ITO/glass) substrate was fabricated by rf magnetron sputtering. The samples were cleaned by acetone, methanol, and deionized [DI] water under sonication. To prepare an aqueous seed solution, the 0.1 M zinc acetate dihydrate (Zn(CH_3_COO)_2_·2H_2_O) was dissolved in DI water. A sonication process was performed while slowly adding the MWCNT paste which was grown by a thermal chemical vapor deposition method. Then, the ITO/glass substrate was spin-coated with this seed solution at 3,000 rpm for 90 s and dried at a hot plate of 80°C for 10 min. After the spin-coating-and-drying procedure was repeated successively five times for a uniform seed layer coating, the sample was heated at 200°C for 1 h to increase the adhesion between the seed layer containing MWCNTs and the substrate. In order to electrochemically grow the ZnO NRAs, the seed layer-coated ITO/glass and platinum [Pt] electrode were immersed into the electrolyte solution containing 2 mM zinc nitrate hexahydrate (Zn(NO_3_)_2_·6H_2_O), 2 mM hexamethylenetetramine (C_6_H_12_N_4_), and DI water. During the electrochemical growth, the temperature of the electrolyte solution and the applied cathodic voltage were kept at 80°C and -2 V, respectively. After the synthesis of ZnO NRAs, the sample was rinsed with flowing DI water and dried by flowing nitrogen gas.

The morphology and structural properties of the fabricated samples were analyzed using a field-emission scanning electron microscope [FE-SEM] (LEO SUPRA 55, Carl Zeiss, Oberkochen, Baden-Württemberg, Germany) with an operating voltage of 15 kV. To prevent or reduce the electric charge accumulation, the samples were coated by Pt sputtering. The orientation and crystallinity of the samples were characterized using an X-ray diffractometer (M18XHF-SRA, Mac Science, Yokohama, Japan) with a monochromated Cu Kα line source (*λ *= 0.154178 nm). The photoluminescence [PL] measurements were performed using a PL mapping system (RPM 2000, Accent Optics, Denver, CO, USA) with a laser source emitting at the wavelength of 266 nm at room temperature. The macroscopic surface property on wettability was characterized from the measurement of the contact angles with the water droplet on the surface of the samples using a contact angle measurement system (Phoenix-300, SEO Co., Ltd., Gyeonggi-do, South Korea) with a measurement accuracy of ± 0.1°.

## Results and discussion

**Figure 1:**
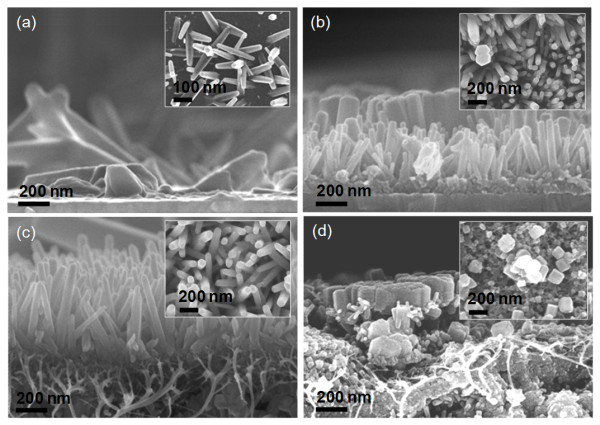
**Cross-sectional and top-view SEM images of the samples**. Cross-sectional SEM images of the electrochemically synthesized (**a**) ZnO nanorods on ITO/glass without the seed layer, (**b**) ZnO NRAs/zinc acetate seed layer on ITO/glass, (**c**) ZnO NRAs/MWCNT (2 wt.%)-composed seed layer on ITO/glass, and (**d**) ZnO NRAs/MWCNT (5 wt.%)-composed seed layer on ITO/glass. The insets show the top-view SEM images of the corresponding samples.

Figure 1 shows the cross-sectional SEM images of the electrochemically synthesized (a) ZnO nanorods on ITO/glass without the seed layer, (b) ZnO NRAs on the zinc acetate seed layer coated on ITO/glass (i.e., ZnO NRAs/seed layer on ITO/glass), (c) ZnO NRAs/MWCNT (2 wt.%)-composed seed layer on ITO/glass, and (d) ZnO NRAs/MWCNT (5 wt.%)-composed seed layer on ITO/glass. The insets of Figure 1 show the top-view SEM images of the corresponding samples. When the ZnO nanorods were synthesized on ITO/glass without the seed layer, they were sparsely populated and randomly orientated on the bare ITO surface. As shown in Figure 1a, ZnO nanorods with no preferred orientation were observed. In contrast, the use of the seed layer gives rise to the ZnO NRAs aligned along a dominant c-axis orientation of the wurzite structure as can be seen in Figure 1b,c,d, which indicates that the spin-coated seed layer can efficiently provide the nucleation sites on the ITO surface [11]. For ZnO NRAs/zinc acetate seed layer on ITO/glass, the ZnO nanorods were densely assembled, but their height and size were not uniformly distributed and at approximately 100 to 800 nm and 20 to 150 nm, respectively. Whereas, the ZnO NRAs/MWCNT (2 wt.%)-composed seed layer on ITO/glass exhibited a more uniform distribution in the height and size. The height and size were about 600 to 1,000 nm and 20 to 50 nm, respectively. It is noted that the ZnO nanorods were more densely and uniformly grown using the MWCNT (2 wt.%)-composed seed layer. This is probably attributed to a uniform electric field between the sample surface and electrolyte solution by the enhanced electrical conductivity of the seed layer. Herein, the electric field plays a key role in the chemical synthesis. It can be expected to improve the electrical and optical properties of ZnO NRAs by forming the ZnO/MWCNT hybrid nanostructure. However, the MWCNT (5 wt.%)-composed seed layer made the ZnO nanostructures shorter and thicker, which creates somewhat aggregated ZnO/MWCNT composites as shown in Figure 1d. This means that such excess MWCNTs in the composed seed layer may prevent the successful formation of ZnO nanorods.

**Figure 2:**
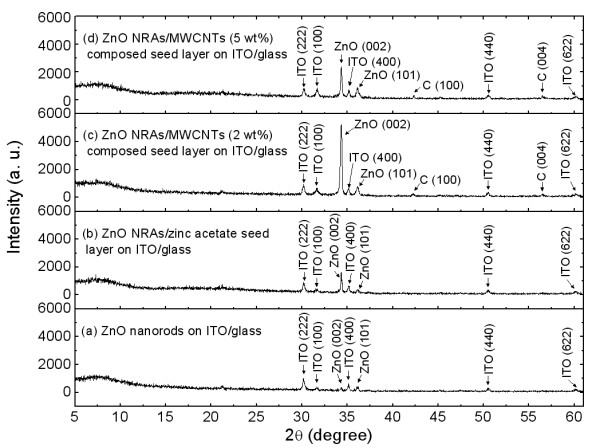
**2*θ *scan XRD patterns of the samples**. 2*θ *scan XRD patterns of the (**a**) ZnO nanorods with no seed layer, (**b**) ZnO NRAs/zinc acetate seed layer, (**c**) ZnO NRAs/MWCNT (2 wt.%)-composed seed layer, and (**d**) ZnO NRAs/MWCNT (5 wt.%)-composed seed layer on ITO/glass.

Figure 2 shows the 2*θ *scan X-ray diffraction [XRD] patterns of the (a) ZnO nanorods with no seed layer, (b) ZnO NRAs zinc acetate seed layer, (c) ZnO NRAs/MWCNT (2 wt.%)-composed seed layer, and (d) ZnO NRAs/MWCNT (5 wt.%)-composed seed layer on ITO/glass. From the XRD patterns, the (100), (222), (400), (440), and (622) XRD peaks of ITO were clearly observed, and they exhibited almost similar intensities for all the samples. For ZnO nanorods grown directly on ITO/glass, the weak peak intensity of ZnO was observed at the (002) and (101) planes due to the poor orientation and low density in Figure 1d. For the ZnO NRAs in Figure 2b,c,d, the XRD peak intensity at the (002) plane of ZnO was increased. This confirms that the spin-coated seed layer enables the crystallization of ZnO nanorods with a hexagonal wurtzite structure and a preferred orientation along the c-axis as mentioned in Figure 1. For ZnO NRAs/MWCNT-composed seed layer as shown in Figure 2c,d, the (100) and (004) XRD peaks of carbon were also observed. By incorporating the MWCNTs into the zinc acetate seed layer, the (002) XRD peak intensity of ZnO was largely enhanced for 2 wt.% MWCNTs, but it was decreased for 5 wt.% MWCNTs. It is clear that the MWCNT-composed seed layer enhances the crystallinity of ZnO nanorods, but the aggregated ZnO/MWCNT composites synthesized on the excess MWCNT-composed seed layer have a degraded crystallinity.

**Figure 3:**
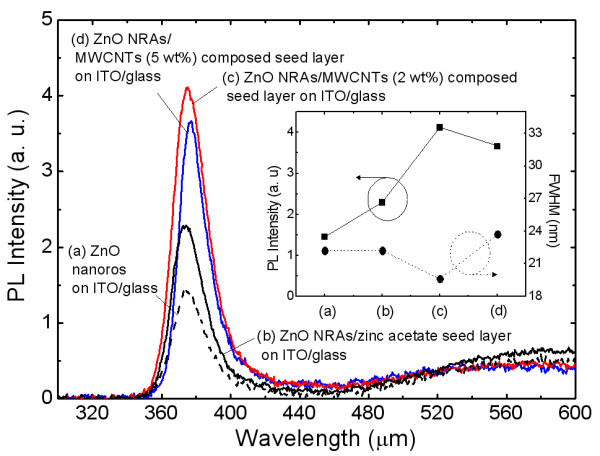
**Room-temperature PL spectra, PL intensity, and FWHM value of the samples**. Room-temperature PL spectra of the (**a**) ZnO nanorods with no seed layer, (**b**) ZnO NRAs/zinc acetate seed layer, (**c**) ZnO NRAs/MWCNT (2 wt.%)-composed seed layer, and (**d**) ZnO NRAs/MWCNT (5 wt.%)-composed seed layer on ITO/glass. The inset shows the PL intensity and the FWHM value of the corresponding samples.

Figure 3 shows the room-temperature PL spectra of the (a) ZnO nanorods with no seed layer, (b) ZnO NRAs/zinc acetate seed layer, (c) ZnO NRAs/MWCNT (2 wt.%)-composed seed layer, and (d) ZnO NRAs/MWCNT (5 wt.%)-composed seed layer on ITO/glass. The inset shows the PL intensity and the full width at half maximum [FWHM] value of the corresponding samples. The peak positions of the near-band-edge [NBE] UV emission for the synthesized ZnO nanorods were observed at wavelengths of 373 to 375 nm. The PL peak intensity of the NBE UV emission was relatively low. The weak and broad visible emissions are related to the structural defects in ZnO nanostructures. For ZnO NRAs with the seed layer, the PL peak intensity of the NBE UV emission was increased, which exhibits a similar tendency with the XRD data. This indicates that the optical property of ZnO nanostructures is also strongly dependent on the seed layer. For the ZnO NRAs/MWCNT (2 wt.%)-composed seed layer, the PL peak intensity was significantly increased, and the NBE UV emission became somewhat more sharp. The PL peak intensity was enhanced by 2.8 times, and a narrow FWHM value of 19.6 nm was obtained compared to the ZnO nanorods grown without the seed layer as shown in the inset of Figure 3. However, when the concentration of MWCNTs in the composed seed layer was increased to 5 wt.%, the structure exhibited an increased FWHM value as well as a reduced PL peak intensity. The enhanced PL peak intensity of the NBE UV emission would be caused by the high crystallinity of ZnO NRAs and surface plasmon-meditated emission enhancement by MWCNTs. Since the estimated surface plasmon energy is about 3.1 to 3.3 eV for the dielectric constant of ZnO, the NBE UV emission of ZnO NRAs may be coupled with the surface plasmon resonance of the CNT [10,12].

**Figure 4:**
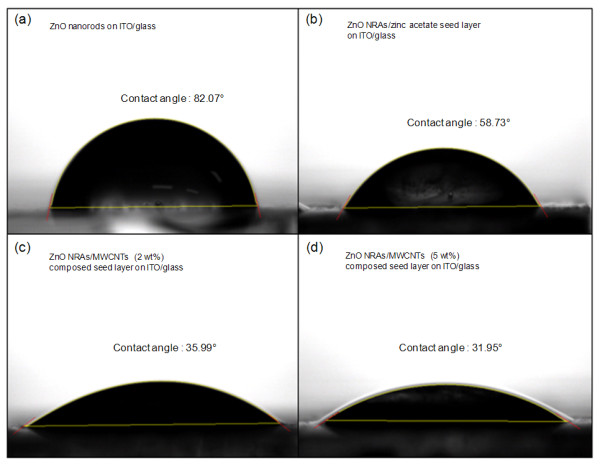
**Photographic images of the water droplet on the surface and contact angles of the samples**. Photographic images of the water droplet on the surface of (**a**) ZnO nanorods with no seed layer, (**b**) ZnO NRAs/zinc acetate seed layer, (**c**) ZnO NRAs/MWCNT (2 wt.%)-composed seed layer, and (**d**) ZnO NRAs/MWCNT (5 wt.%)-composed seed layer on ITO/glass and the measured contact angles of the corresponding samples.

Figure 4 shows the photographic images of the water droplet on the surface of (a) ZnO nanorods with no seed layer, (b) ZnO NRAs/zinc acetate seed layer, (c) ZnO NRAs/MWCNT (2 wt.%)-composed seed layer, and (d) ZnO NRAs/MWCNT (5 wt.%)-composed seed layer on ITO/glass. The measured contact angles of the corresponding samples are also shown. The ZnO nanorods grown directly on ITO/glass exhibited a surface wettability with a contact angle of 82.07° because the ZnO nanorods were sparsely distributed and not well aligned. It is noticeable that the surface of ZnO is known to be hydrophilic [13]. For ZnO NRAs with the seed layer, however, the contact angle was gradually decreased as 58.73°, 35.99°, and 31.95°, as can be seen in Figure 4b,c,d, respectively. The surface macroscopic property on the wettability became more hydrophilic for high-density, ordered ZnO NRAs. Therefore, the surface-modified structure with a better hydrophilic property can be expected to be used for microfluidic device applications.

## Conclusion

The structural and optical properties of electrochemically synthesized ZnO NRAs on the ITO/glass substrate using MWCNT-composed seed layers formed by a simple method were investigated. It was found that the morphology of ZnO NRAs strongly depends on the kinds of seed layers. The optimized MWCNT-composed seed layer resulted in the high-density, well-aligned ZnO NRAs with a high crystallinity. The PL peak intensity of the NBE UV emission in ZnO/MWCNT hybrid nanostructures was significantly increased due to the surface plasmon-meditated emission enhancement by MWCNTs as well as the improved crystallization property. Also, the surface macroscopic property on the wettability could be modified with more hydrophilic characteristics. This simple electrochemical fabrication method using the seed layer containing CNTs is very useful to grow high-quality ZnO NRAs on an ITO/glass substrate for various optoelectronic device applications.

## Competing interests

The authors declare that they have no competing interests.

## Authors' contributions

YHK designed and analyzed the composed nanostructures by performing the measurements (FE-SEM, XRD, PL, contact angles), and MSK assisted in synthesizing the composed seed layer, growing the nanostructure, and analyzing each sample. The experiment and the writing of the manuscript were carried out under the instruction of JSY. All authors read and approved the final manuscript.
